# Preoperative Skeletal Muscle Index as an Independent Predictor of Anastomotic Leakage After Colorectal Cancer Surgery: A Retrospective Cohort Study

**DOI:** 10.3390/diagnostics16162581

**Published:** 2026-08-15

**Authors:** Mehmet Baykan, Tuba Yücel Uçarkuş, Gökçe Ersolak, İsmail Altintop

**Affiliations:** 1 Department of General Surgery, Kayseri City Hospital, 38080 Kayseri, Türkiye; gokceersolak@gmail.com; 2 Department of Radiology, Kayseri City Hospital, 38080 Kayseri, Türkiye; drtubayucel@gmail.com; 3 Department of Emergency Medicine, Kayseri Faculty of Medicine, University of Health Sciences, 38080 Kayseri, Türkiye

**Keywords:** colorectal cancer, anastomotic leakage, sarcopenia, skeletal muscle index, computed tomography, body composition, surgical risk stratification

## Abstract

**Background:** Anastomotic leakage remains the most feared complication after colorectal cancer surgery, and reliable preoperative risk stratification is still evolving. The skeletal muscle index (SMI), derived from routine computed tomography, offers an objective, widely available measure of muscle mass. This study evaluated whether a low preoperative SMI predicts anastomotic leakage after colorectal cancer resection. **Methods:** We retrospectively screened 550 consecutive patients who underwent colorectal cancer resection with primary anastomosis at a single tertiary referral centre between 2018 and 2024, of whom 468 satisfied the eligibility criteria and formed the analytic cohort. Skeletal muscle area was measured on preoperative axial computed tomography at the third lumbar vertebra and normalised to the height squared to obtain the SMI. Anastomotic leakage was defined according to the International Study Group of Rectal Cancer criteria. Receiver operating characteristic analysis identified a single pragmatic SMI cut-off. Group comparisons used Mann–Whitney U and chi-square tests, and independent predictors were assessed by multivariable logistic regression. **Results:** Anastomotic leakage occurred in 33 of 468 patients (7.1%). Patients who developed leakage had a significantly lower mean SMI than those who did not (40.0 cm^2^/m^2^ vs. 46.8 cm^2^/m^2^, *p* = 0.005). An SMI below 48.6 cm2/m2 predicted leakage with 90.9% sensitivity, 38.2% specificity, and a 98.2% negative predictive value (area under the curve 0.646). Because specificity (38.2%) and positive predictive value (10.0%) were low, the index performed as a rule-out marker: a preserved SMI identified a low-risk subgroup, whereas a low-SMI had limited positive predictive value. The leakage rate reached 10.0% in the low SMI group versus 1.8% in the high-SMI group (*p* = 0.0008). Following multivariable analysis, low SMI was the only independent predictor of anastomotic leakage (adjusted odds ratio: 5.67, 95% confidence interval: 1.69 to 19.01, *p* = 0.005). **Conclusions:** A low preoperative SMI independently increased the odds of anastomotic leakage almost six-fold after colorectal cancer surgery, while its high negative predictive value identified a large group of patients at very low risk. Routine SMI assessment on existing staging computed tomography may strengthen preoperative risk stratification at no additional cost or radiation.

## 1. Introduction

Colorectal cancer ranks among the most common malignancies worldwide and remains one of the leading causes of cancer related death. Recent global estimates place it third in incidence and second in cancer related mortality, with close to two million new cases reported each year [1,2]. For early-stage and resectable disease, surgical resection forms the cornerstone of curative treatment. The oncological benefit of resection, however, must always be weighed against the risks of postoperative complications, which can compromise both the recovery and the long-term prognosis of the patient [2,3].

Among these complications, anastomotic leakage stands out as the most serious. Leakage is defined as a communication between the intraluminal and extraluminal compartments at the anastomotic site, and it can progress to a localised infection, a pelvic abscess, peritonitis, sepsis, reoperation, and a prolonged hospital stay [3,4]. The reported incidence varies considerably across series because of the differences in patient characteristics, tumour location, surgical technique, and diagnostic criteria applied [3,5]. Beyond its immediate morbidity and mortality, anastomotic leakage has been linked to higher local recurrence rates and reduced cancer-specific survival, which makes its prevention a priority that extends well past the perioperative window.

The pathogenesis of anastomotic leakage is multifactorial, and patient-related, tumour-related, and surgical factors all contribute. Male sex, advanced age, obesity, smoking, comorbidity, malnutrition, hypoalbuminaemia, neoadjuvant therapy, distal tumour location, and low anastomotic levels have all been described as risk factors [3,5]. Intraoperative determinants, such as anastomotic perfusion, tension, blood loss, operative time, surgical approach, anastomotic technique, and experience of the surgeon, further modulate the risk [3,5]. Despite this extensive list, there remains an unmet clinical need for a simple, objective, and reproducible preoperative marker that reliably identifies patients at the highest risk of leakage.

Over the past decade, body composition parameters have attracted growing attention as candidate predictors of surgical outcomes. Sarcopenia, traditionally defined as the progressive and generalised loss of skeletal muscle mass and function, sits at the centre of this interest [6,7]. The skeletal muscle index (SMI) provides an objective estimate of muscle mass and is most often derived from a single axial computed tomography image at the level of the third lumbar vertebra, where the muscle area correlates closely with whole-body muscle mass. The cross-sectional muscle area is then normalised to the square of the patient height [6,8]. Because almost every patient with colorectal cancer undergoes staging computed tomography, the SMI can be obtained from existing images without additional cost, contrast, or radiation.

A low SMI has been associated with frailty, malnutrition, a blunted systemic anabolic response, and impaired tissue healing, all of which are biologically plausible mechanisms for anastomotic failure [9,10,11]. Several investigators have reported associations between low muscle mass and adverse outcomes after gastrointestinal surgery, yet the evidence specific to anastomotic leakage in colorectal cancer remains heterogeneous, and many studies were limited by small samples, mixed muscle measurement methods or a focus on overall complications rather than leakage itself [11,12,13]. Against this background, the present study evaluated the relationship between the preoperative SMI measured on routine computed tomography and the occurrence of anastomotic leakage in a large single centre cohort of patients with colorectal cancer, and tested whether a low SMI behaves as an independent predictor of leakage after adjustment for established clinical covariates.

## 2. Materials and Methods

### 2.1. Study Design and Population

We conducted a retrospective cohort study by screening 550 consecutive patients who underwent elective resection for histologically confirmed colorectal cancer with primary anastomosis at Kayseri City Hospital between 2018 and 2024. The study was reported in accordance with the Strengthening the Reporting of Observational Studies in Epidemiology (STROBE) statement [14]. The study protocol conformed to the principles of the Declaration of Helsinki, and approval was obtained from the institutional non-interventional clinical research ethics committee of Kayseri City Hospital (protocol code: 170, date of approval: 10 September 2024). Because of the retrospective design and the use of de-identified data, the requirement for written informed consent was waived.

**Figure 1:**
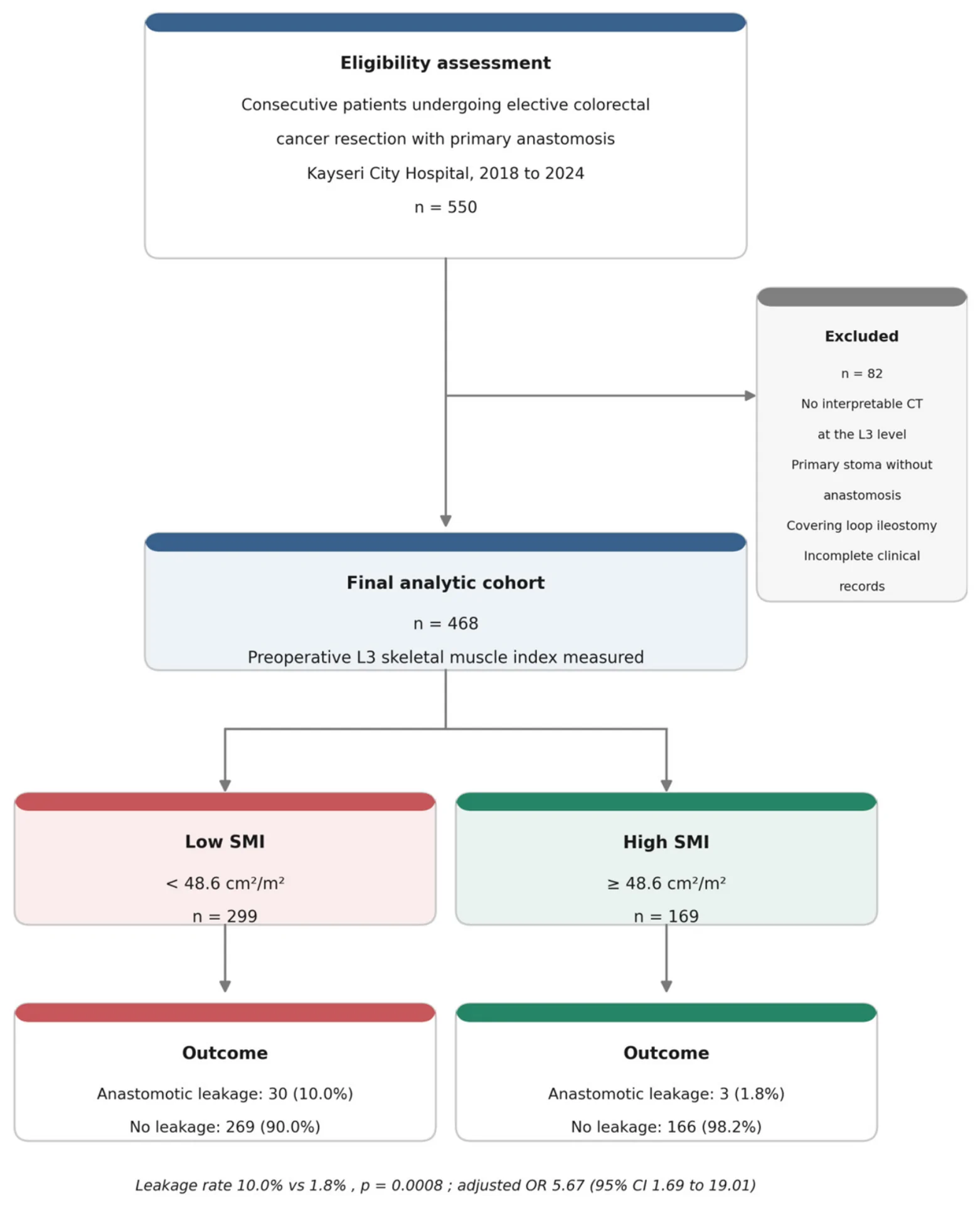
**Study flow diagram.** A total of 550 consecutive patients undergoing elective colorectal cancer resection with primary anastomosis at Kayseri City Hospital between 2018 and 2024 were assessed for eligibility, and 82 were excluded. The final analytic cohort of 468 patients was stratified by the preoperative skeletal muscle index, and the anastomotic leakage outcome is shown within each group.

Adult patients (aged 18 years or older) with a preoperative abdominal computed tomography examination of adequate quality at the third lumbar vertebral level and a primary anastomosis were eligible. Patients without an interpretable computed tomography study at the required level, those who received a primary stoma without anastomosis, and those with incomplete records were excluded. Patients whose anastomosis was protected by a covering loop ileostomy were also excluded, so that leakage was assessed only in undiverted anastomoses in which a leak would be neither diverted nor clinically masked. Consequently, out of the initial 550 screened cases, 82 patients were excluded and 468 patients satisfied these eligibility criteria, forming the final analytic cohort. The study flow, including the eligibility assessment, the stratification by skeletal muscle index, and the leakage outcome within each group, is summarised in Figure 1.

### 2.2. Computed Tomography Acquisition and Skeletal Muscle Analysis

All computed tomography examinations were performed using a 128-slice multidetector scanner (Somatom Definition AS, Siemens Healthineers, Erlangen, Germany). Skeletal muscle segmentation was performed on a dedicated post-processing workstation using syngo.via (version VB60; Siemens Healthineers, Erlangen, Germany). At the third lumbar level, the skeletal muscle compartments, including the psoas, the paraspinal, and the abdominal wall muscles, were manually delineated by an experienced investigator who was strictly blinded to the clinical outcomes and postoperative complications of the patients. After manual contouring, the software identified skeletal muscle tissue automatically using its predefined attenuation-based segmentation algorithm within a Hounsfield Unit (HU) threshold range of −29 to +150 HU. The skeletal muscle area was computed automatically and recorded in square centimetres.

The skeletal muscle index was then calculated by normalising the skeletal muscle area to the square of the patient height, according to the formula SMI (cm^2^/m^2^) = skeletal muscle area (cm^2^) divided by height squared (m^2^). All measurements were performed by a single abdominal radiologist (with 20 years of experience in cross-sectional imaging) who was strictly blinded to the patients’ clinical outcomes and postoperative complication status. This approach ensured high consistency across the cohort and successfully removed inter-observer variability. The measured volume, section thickness, and segmented muscle surface area were recorded for every patient, together with the patient height, weight, and derived body mass index. Lean tissue imaging at the third lumbar level on cross-sectional computed tomography is an established and reproducible method for objective muscle and nutritional assessment [15].

### 2.3. Outcome Definition

The primary outcome was anastomotic leakage, defined according to the criteria of the International Study Group of Rectal Cancer (ISGRC) as a defect of the intestinal wall at the anastomotic site that produces a communication between the intraluminal and extraluminal compartments, including pelvic abscesses in proximity to the anastomosis [3]. Leakage was confirmed by clinical, radiological, or operative findings during the index admission and within the first 30 days of the postoperative period. Patients were classified into two groups according to whether anastomotic leakage was present.

### 2.4. Statistical Analysis

Statistical analyses were performed with IBM SPSS Statistics version 25.0 (IBM Corp., Armonk, NY, USA). Categorical variables are presented as counts and percentages, and continuous variables as mean with standard deviation, supplemented by the median with the minimum and maximum where appropriate. The Kolmogorov–Smirnov test assessed the normality of the distribution of continuous variables. Because the relevant continuous parameters departed from a normal distribution, the Mann–Whitney U test was used for between-group comparisons of continuous variables, and the chi-square test or Fisher’s exact test, where appropriate, was used for categorical comparisons.

The discriminative ability of the SMI for anastomotic leakage was evaluated by receiver operating characteristic analysis. The area under the curve with its 95% confidence interval was calculated, and a data-derived threshold was identified by maximising the Youden index; this internally derived value is exploratory and is not proposed as a clinically established cut-off. At the selected thresholds, sensitivity, specificity, positive predictive values, and negative predictive values were derived. Patients were then dichotomised into a low-SMI group and a high-SMI group according to this cut-off. Independent predictors of anastomotic leakage were identified by multivariable binary logistic regression using a backward elimination method, which incorporated the dichotomised SMI with body mass index, sex, and age as established clinical covariates. Odds ratios with 95% confidence intervals are reported. A two-sided *p* value below 0.05 was considered statistically significant. The figures were prepared with Python version 3.11, using the Matplotlib (version 3.8) and scikit-learn (version 1.4) libraries, and reproduced the primary SPSS output.

## 3. Results

### 3.1. Baseline Characteristics of the Cohort

The analytic cohort comprised 468 patients, of whom 33 (7.1%) developed anastomotic leakage and 435 (92.9%) did not. Men made up the majority of the cohort, accounting for 299 patients (63.9%), while 169 patients (36.1%) were women. The mean age was 63.9 (standard deviation 13.3) years, with a median of 66 years and a range of 22 to 101 years. The mean height was 168.0 (7.1) cm, and the mean body mass index was 26.9 (3.8) kg/m^2^, which placed the cohort, on average, within the overweight category. The mean SMI was 46.4 (11.1) cm^2^/m^2^, with a median of 44.2 cm^2^/m^2^. The mean segmented muscle surface area was 127.6 (35.0) cm^2^, the mean measured volume was 22.1 (17.4) cm^3^, and the mean section thickness was 1.65 (1.1) mm. The baseline characteristics of the cohort are summarised in Table 1.

### 3.2. Representative Skeletal Muscle Segmentation

**Figure 2:**
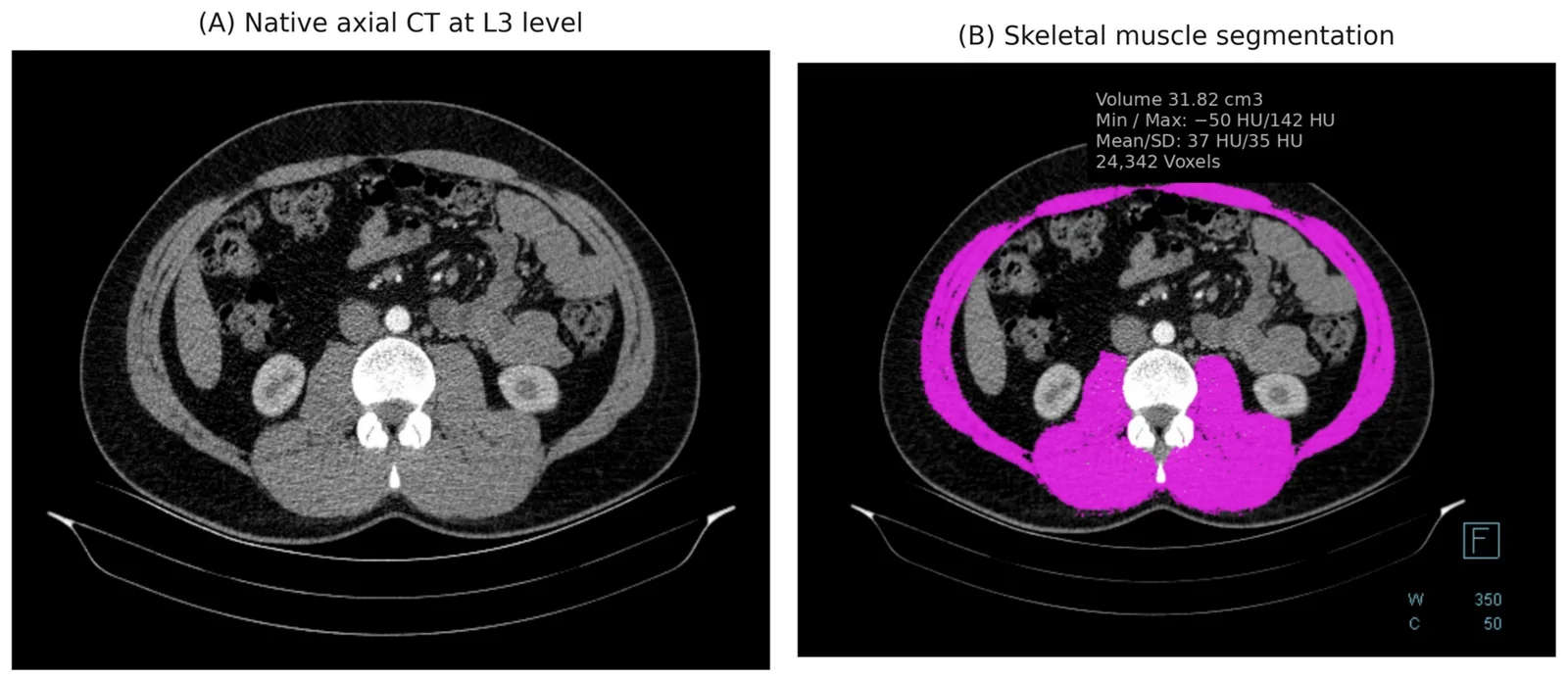
**Representative skeletal muscle segmentation at the third lumbar vertebra.** (**A**) Native axial computed tomography image at the level of the third lumbar vertebra. (**B**) Skeletal muscle compartments, including the psoas, paraspinal, and abdominal wall muscles, segmented with attenuation-based tissue identification within a Hounsfield Unit threshold range of −29 to +150 HU. The segmented muscle area was normalised to the height squared to obtain the skeletal muscle index.

Figure 2 illustrates the skeletal muscle segmentation method applied to every patient. The native axial computed tomography image at the third lumbar vertebra is shown alongside the corresponding segmented image, in which the psoas, paraspinal, and abdominal wall muscle compartments are highlighted after attenuation-based tissue identification. This reproducible workflow yielded the skeletal muscle area from which the SMI was derived, and it relied only on images already acquired for routine oncological staging.

### 3.3. Discriminative Performance of the Skeletal Muscle Index

**Figure 3:**
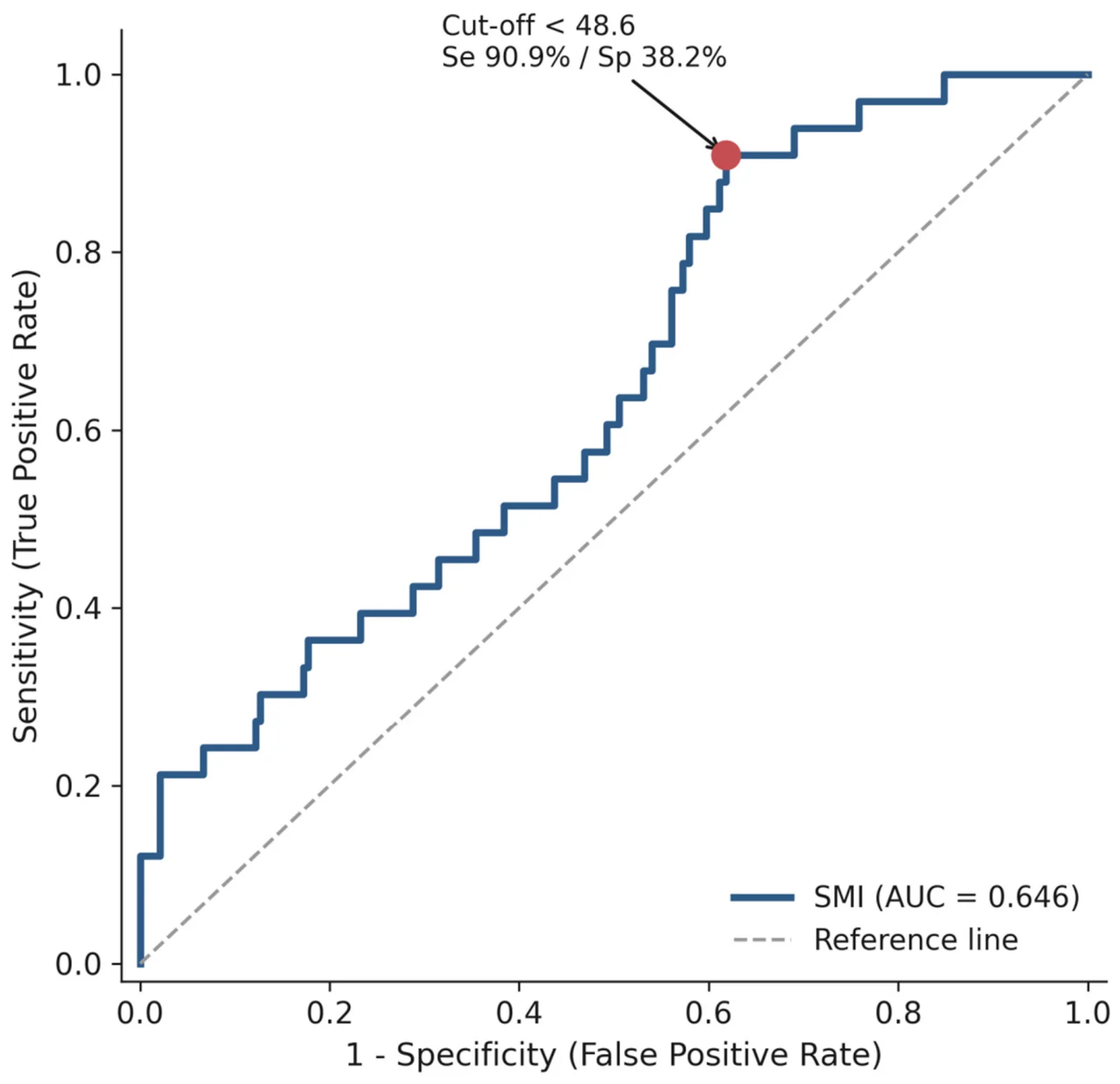
**Receiver operating characteristic curve of the skeletal muscle index for the prediction of anastomotic leakage.** The area under the curve was 0.646. The red marker denotes the data-derived threshold (skeletal muscle index below 48.6 cm^2^/m^2^) identified by the Youden index, with a sensitivity of 90.9% and a specificity of 38.2%.

Figure 3 presents the receiver operating characteristic curve for the SMI in the prediction of anastomotic leakage. The area under the curve was 0.646 (95% confidence interval 0.599 to 0.688), which indicates a modest but statistically significant discriminative ability (*p* = 0.002). The Youden index identified a data-derived SMI threshold of 48.6 cm^2^/m^2^. At this cut-off, a value below 48.6 cm^2^/m^2^ predicted anastomotic leakage with a high screening sensitivity of 90.9% and a specificity of 38.2%. The positive predictive value was 10.0% and the negative predictive value was 98.2%, which means that the great majority of patients with a preserved SMI did not leak. Applying this threshold classified 299 of 468 patients (63.9%) as low index, yet only 30 of these developed leakage, so approximately nine out of every ten patients flagged did not experience leakage. The threshold therefore performs as a rule-out rather than a rule-in tool, with its value lying in identifying the preserved-index subgroup at low risk rather than in predicting which individual patient will leak. The high negative predictive value should be interpreted with caution, as it partly reflects the low overall leakage prevalence (7.1%) rather than strong discrimination. The full diagnostic performance metrics are detailed in Table 2.

A sex-stratified analysis was performed to assess whether a single threshold was appropriate. The skeletal muscle index was lower in women than in men (43.6 ± 10.7 versus 47.9 ± 11.0 cm^2^/m^2^). Sex-specific thresholds were close to the pooled value (men, SMI < 48.6 cm^2^/m^2^, area under the curve 0.689; women, SMI < 47.8 cm^2^/m^2^, area under the curve 0.567), and discrimination was weaker among women. Applying sex-specific thresholds classified 293 patients as low index with a leakage rate of 9.6%, compared to 2.9% in the high-index group, results which were comparable to those obtained with the single threshold.

### 3.4. Skeletal Muscle Index by Leakage Status

**Figure 4:**
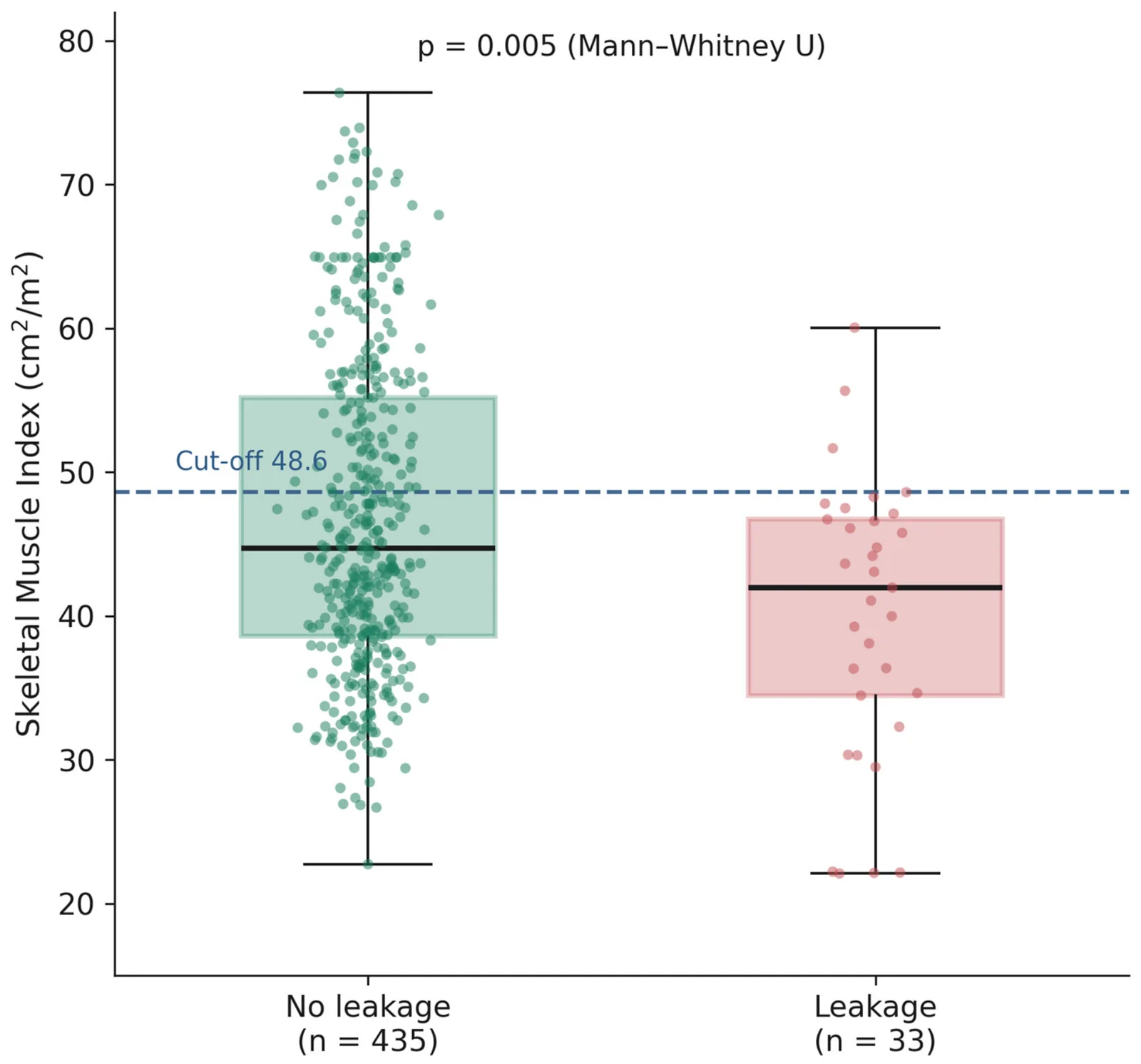
**Distribution of the skeletal muscle index by anastomotic leakage status.** Box plots with individual data points show a significantly lower skeletal muscle index in patients who developed anastomotic leakage (*p* = 0.005, Mann–Whitney U test). The dashed line marks the cut-off of 48.6 cm^2^/m^2^.

Figure 4 compares the distribution of SMI between patients who developed anastomotic leakage and those who did not. Patients with leakage had a markedly lower SMI, with a mean of 40.0 ± 9.8 cm^2^/m^2^ (median: 42.0 cm^2^/m^2^), compared to a mean of 46.8 ± 11.1 cm^2^/m^2^ (median: 44.7 cm^2^/m^2^) in patients without leakage. This difference was statistically significant on the Mann–Whitney U test (*p* = 0.005). The displacement of the leakage group below the 48.6 cm^2^/m^2^ threshold provides a visual counterpart to the regression findings reported below.

### 3.5. Anastomotic Leakage Rate Across Skeletal Muscle Index Groups

**Figure 5:**
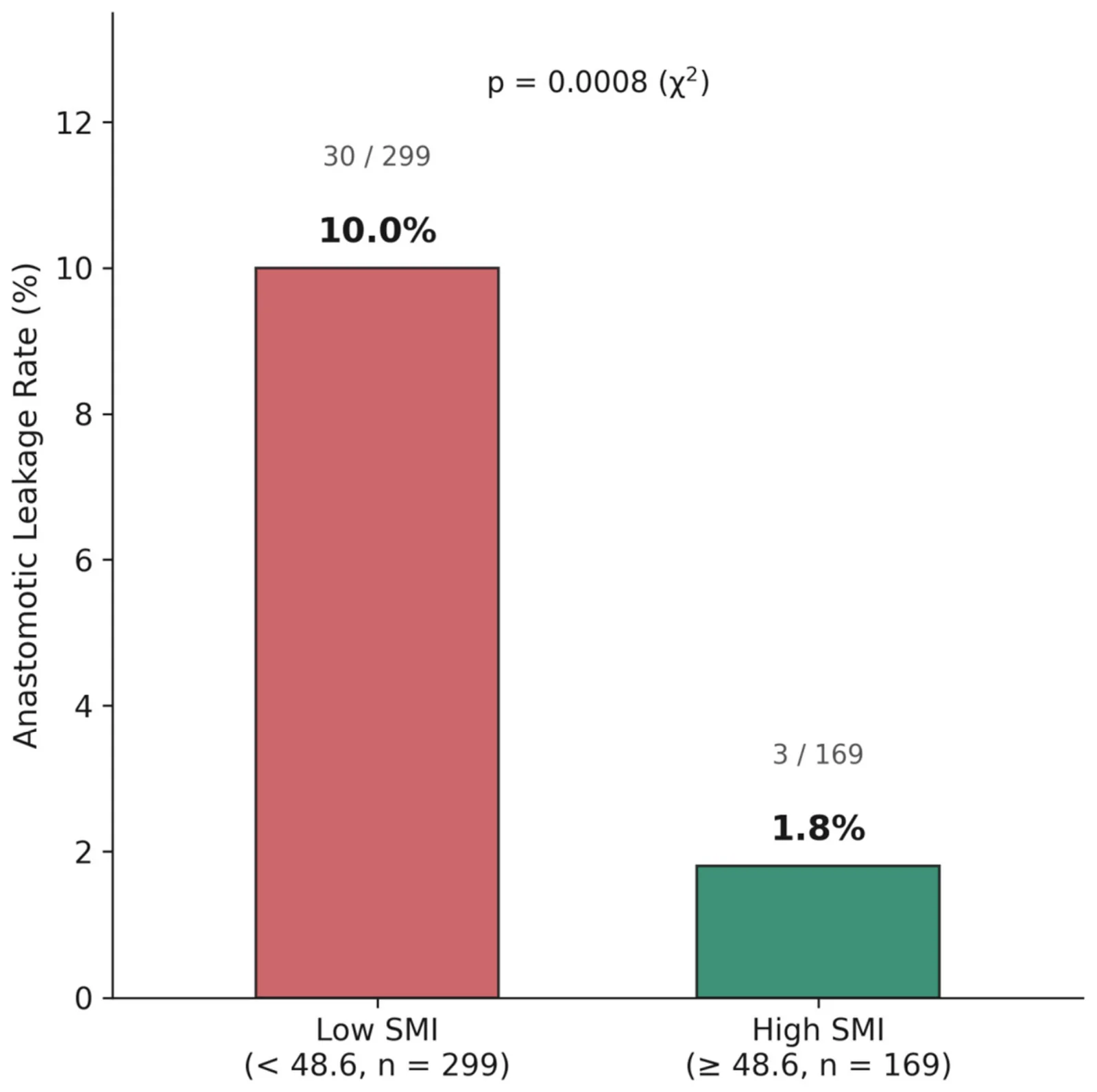
**Anastomotic leakage rate stratified by skeletal muscle index group.** Leakage occurred in 10.0% of patients with a low skeletal muscle index (below 48.6 cm^2^/m^2^) versus 1.8% of patients with a high skeletal muscle index (*p* = 0.0008, chi-square test).

Figure 5 displays the anastomotic leakage rate within the two SMI groups defined by the 48.6 cm^2^/m^2^ cut-off. Leakage developed in 30 of 299 patients in the low-SMI group, a rate of 10.0%, and in only three of 169 patients in the high-SMI group, a rate of 1.8%. This more than five-fold difference in absolute leakage rate was highly significant on the chi-square test (*p* = 0.0008). The contrast highlights how strongly the leakage burden concentrated among patients with depleted muscle mass.

### 3.6. Comparison of Clinical and Body Composition Variables Between Groups

Table 3 compares the demographic, clinical, and body composition parameters between the low- and high-SMI groups. Sex distribution differed significantly between the groups. The high-SMI group was predominantly male, with men comprising 72.8% of the group, whereas the low-SMI group contained a higher proportion of women (41.1% versus 27.2%, *p* = 0.002). This pattern is consistent with the physiological expectation that men carry greater skeletal muscle mass. The leakage rate differed sharply between groups as already described. In contrast, age (*p* = 0.535), body mass index (*p* = 0.196), and height (*p* = 0.178) did not differ significantly between the groups, which indicates that the SMI captured information not reflected by these conventional anthropometric measures. As expected, the radiological measures directly tied to muscle bulk, namely the measured volume, section thickness, and segmented surface area, were all significantly higher in the high-SMI group (*p* < 0.001 for each).

### 3.7. Independent Predictors of Anastomotic Leakage

**Figure 6:**
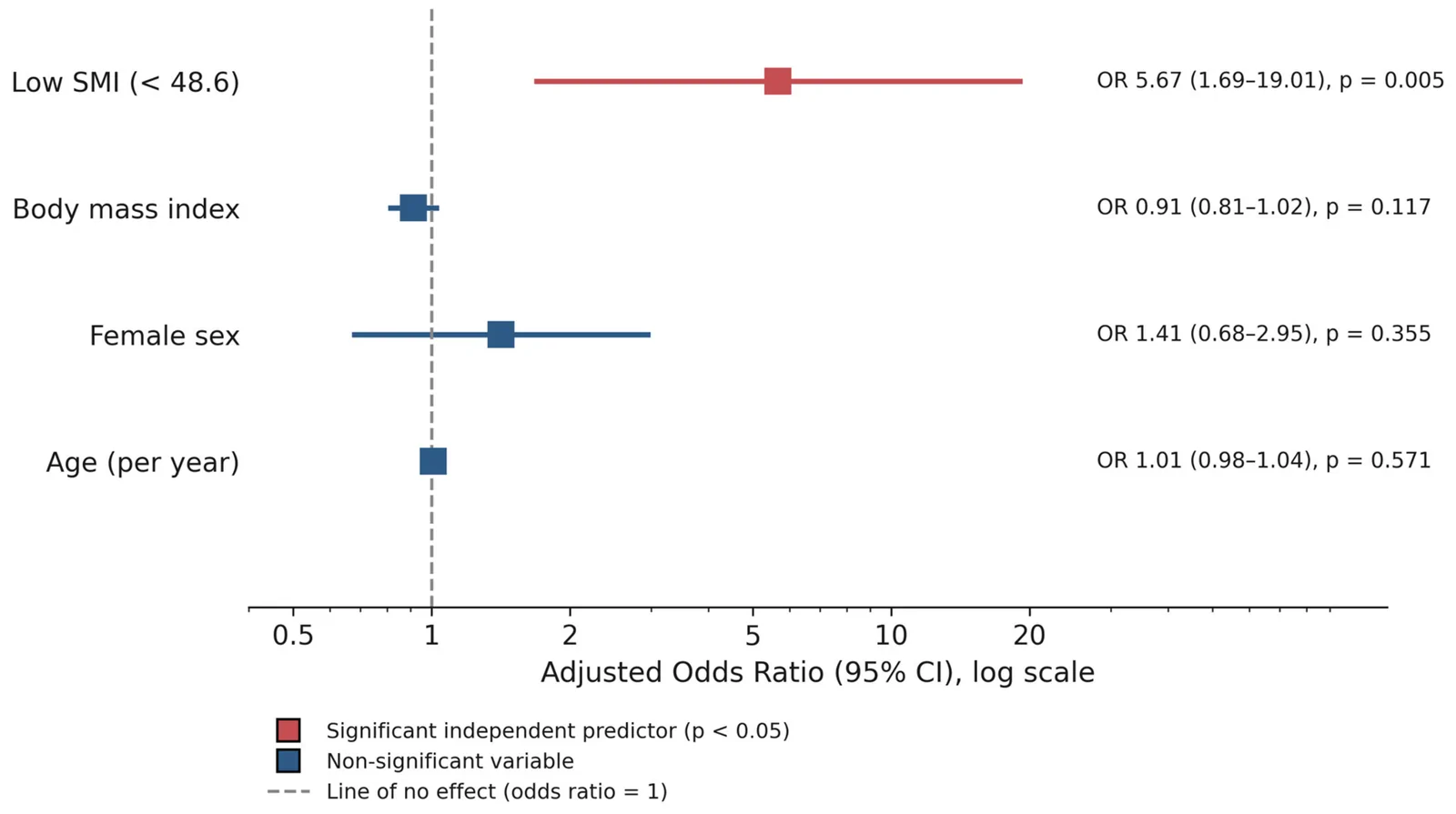
**Forest plot of the multivariable logistic regression for anastomotic leakage.** Adjusted odds ratios with 95% confidence intervals are shown on a logarithmic scale. A low skeletal muscle index was the only independent predictor of anastomotic leakage (odds ratio 5.67, 95% confidence interval 1.69 to 19.01, *p* = 0.005).

Figure 6 and Table 4 present the multivariable logistic regression analysis of factors associated with anastomotic leakage. After simultaneous adjustment for body mass index, sex, and age, a low SMI emerged as the only independent predictor of anastomotic leakage. Patients with an SMI below 48.6 cm^2^/m^2^ had an adjusted odds ratio of 5.67 for leakage (95% confidence interval 1.69 to 19.01, *p* = 0.005). Because the confidence interval lay entirely above unity, this association was statistically robust. None of the remaining covariates reached significance: body mass index (odds ratio 0.91, *p* = 0.117), female sex (odds ratio 1.41, *p* = 0.355), and age (odds ratio 1.01, *p* = 0.571) all showed confidence intervals that crossed unity. The forest plot in Figure 6 visualises these estimates and underscores that a low SMI carried by far the largest and only significant effect.

### 3.8. Body Composition Differences and Sex Distribution

**Figure 7:**
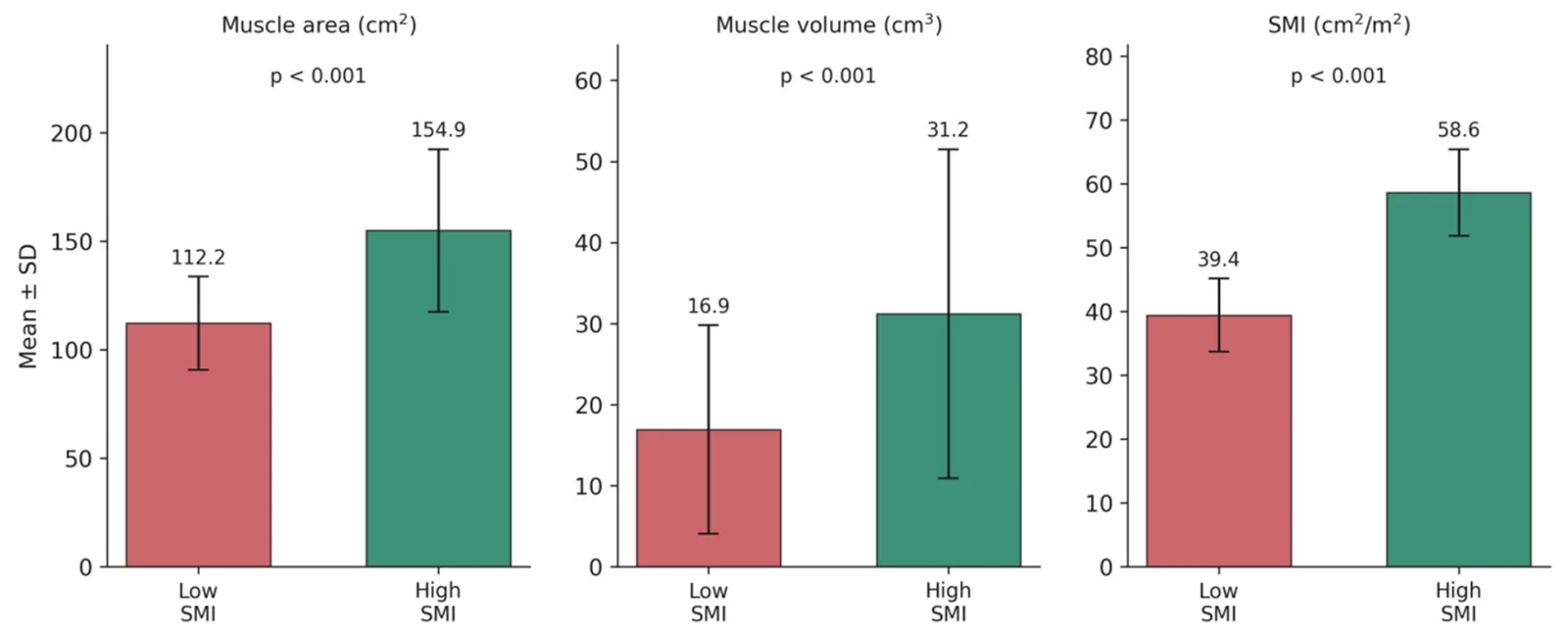
**Comparison of body composition parameters between skeletal muscle index groups.** The muscle surface area, measured muscle volume, and skeletal muscle index were all significantly higher in the high skeletal muscle index group (*p* < 0.001 for each, Mann–Whitney U test). Bars show mean values and error bars show the standard deviation.

Figure 7 contrasts the principal body composition parameters between the two SMI groups and confirms the internal consistency of the segmentation. The muscle surface area, the measured muscle volume, and the SMI itself were all substantially higher in the high-SMI group than in the low-SMI group, with every comparison reaching a *p* value below 0.001. These differences validate the SMI as a faithful summary of the underlying muscle measurements rather than an artefact of normalisation.

**Figure 8:**
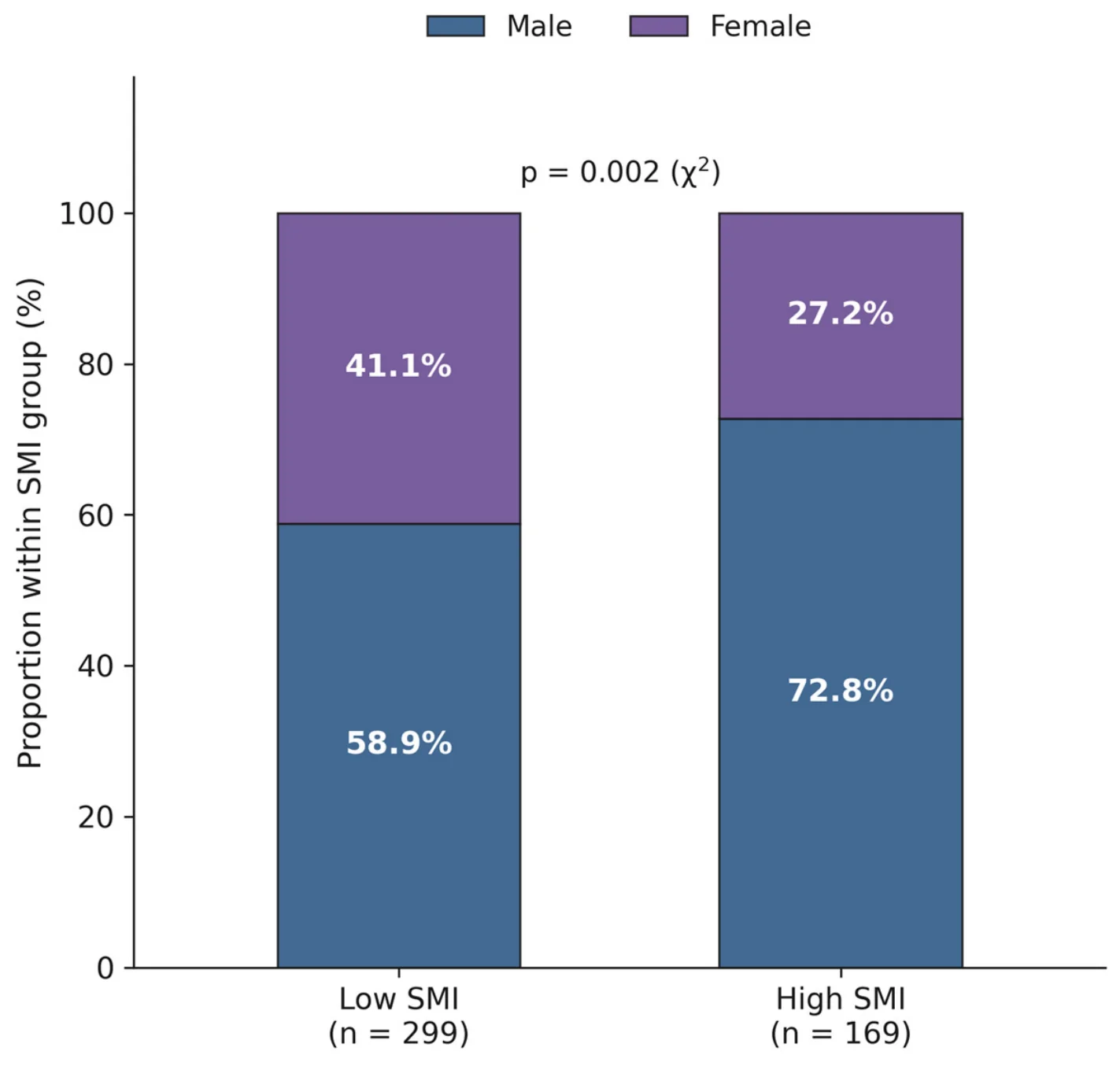
**Sex distribution within the skeletal muscle index groups.** The high skeletal muscle index group was predominantly male, while the proportion of female patients was higher in the low skeletal muscle index group (*p* = 0.002, chi-square test).

Figure 8 depicts the sex composition of the two SMI groups. The high-SMI group predominantly comprised men (72.8%), whereas women were relatively more frequent in the low-SMI group (41.1% versus 27.2%, *p* = 0.002). This distribution reflects established sex differences in muscle mass and reinforces the importance of interpreting the SMI within an appropriate clinical context.

## 4. Discussion

A surgeon can complete a technically flawless anastomosis, confirm a tension-free join, verify a healthy blood supply, and still watch the suture line fail several days later. When that happens, the consequences cascade quickly into sepsis, reoperation, a permanent stoma, and, for some patients, a shortened life. The central question of this study was whether a value that already sits unused inside every staging computed tomography scan can warn the surgeon before the operation begins. Our findings answer that question in the affirmative: a low preoperative SMI (below 48.6 cm^2^/m^2^) independently raised the odds of anastomotic leakage almost six-fold (adjusted odds ratio: 5.67; 95% confidence interval: 1.69 to 19.01; *p* = 0.005), and it did so as the sole significant predictor in a model that also contained body mass index, sex, and age.

In our cohort of 468 patients, anastomotic leakage occurred in 7.1%, a figure that falls squarely within the commonly reported range for colorectal cancer surgery and supports the representativeness of the sample. The leakage group carried a significantly lower SMI than the non-leakage group (mean: 40.0 versus 46.8 cm^2^/m^2^), and the dichotomised analysis sharpened this signal further, with a leakage rate of 10.0% in the low-SMI group against 1.8% in the high-SMI group. The multivariable model then isolated low SMI as the only independent driver of this difference, with an adjusted odds ratio of 5.67. Together, these results position the SMI not as a passive marker of frailty but as an actionable preoperative risk variable that can guide clinical decision-making and patient optimisation.

The biological rationale for this association is coherent. A low SMI is the radiological signature of sarcopenia, which the European and Asian working groups define as the progressive loss of muscle mass and function [6,7,8]. Sarcopenia rarely travels alone. It clusters with malnutrition, a chronic low-grade inflammatory state, impaired protein synthesis, and a diminished capacity for tissue repair, and each of these mechanisms can undermine the delicate process of anastomotic healing [9,10]. Malietzis and colleagues demonstrated that low muscularity and myosteatosis correlate directly with the host systemic inflammatory response in patients undergoing colorectal cancer surgery, which provides a mechanistic bridge between depleted muscle and the breakdown of a fresh anastomosis [16,17]. Crucially, this muscle depletion often occurs covertly beneath a normal or high body mass index (BMI), a phenomenon known as sarcopenic obesity. In our study, despite the stark disparities in anastomotic outcomes and muscle metrics, the mean BMI remained virtually identical between the low- and high-SMI groups (26.7 ± 3.6 vs. 27.2 ± 4.3 kg/m^2^, *p* = 0.196). This clearly demonstrates that conventional anthropometric tools fail to unmask this high-risk patient profile. A muscle-depleted patient is, in effect, a patient whose reparative reserves are already spent before the first incision.

Our findings align with, and substantially extend, a body of evidence that has accumulated rapidly over the past three years. In a 2025 retrospective study in the International Journal of Colorectal Disease, Su and colleagues reported that computed tomography derived sarcopenia tripled the risk of postoperative complications after laparoscopic resection of non-metastatic colorectal cancer, with an odds ratio of 3.42 in a cohort of 387 patients [13]. The 2025 systematic review and meta-analysis by Keshavjee and colleagues, which pooled 40 studies and 13,422 patients, confirmed that sarcopenia worsens postoperative outcomes across colorectal cancer surgery [12]. The 2024 meta-analysis by van Helsdingen and colleagues in the Journal of Cachexia, Sarcopenia and Muscle, which synthesised 45 studies and 16,537 patients, likewise concluded that computed-tomography-derived body composition carries prognostic weight for postoperative complications, although it found the association of muscle measures with leakage weaker than that of visceral fat [11]. The 2025 study by Tirnova and colleagues reached the same broad conclusion in older patients undergoing curative colon resection, where low muscle mass independently predicted major complications [18]. Against this backdrop, our results add a large single-centre cohort in which low muscle mass behaves as a strong and independent predictor of leakage specifically rather than of complications in aggregate. The same direction of effect recurs across gastrointestinal surgical oncology, where large cohorts and meta-analyses have linked low muscle mass to severe postoperative complications and to worse survival in colorectal and gastric cancer [19,20,21,22,23,24].

It is instructive to place our adjusted odds ratio of 5.67 on the spectrum of effect sizes that the recent literature reports for anastomotic leakage. At the upper extreme, Herrod and colleagues found that sarcopenia, defined by psoas density, raised the odds of leakage after colorectal resection with an adjusted odds ratio of 14.37, albeit with a very wide confidence interval (1.37 to 150.04) that betrays a small number of events [25]. Our estimate of 5.67 sits below that figure but well above the values reported in several other recent series. In a 2024 to 2025 multi-centre study of Ivor–Lewis esophagectomy, sarcopenia independently predicted anastomotic leak with an odds ratio of 1.41, and the leak rate doubled in sarcopenic patients (21.6% versus 10.8%). In a 2025 propensity-score-matched analysis of 787 patients undergoing rectal cancer surgery, the sarcopenia group experienced significantly more anastomotic leakage than the non-sarcopenia group (1.9% versus 0%, *p* = 0.008), echoing the direction of effect first described for rectosigmoid resections by van Kooten and colleagues [26]. A 2025 prospective study by Chirivella-Fernandez and colleagues further linked radiologically defined sarcopenia and low folic acid levels to leakage in patients undergoing colorectal resection with anastomosis [27]. The breadth of these estimates, which spans an order of magnitude, is itself an important observation. While the mathematical stability of some historical series is challenged by zero-event cells or extreme confidence intervals, our model provides a statistically balanced intermediate that avoids these extremes. The wide confidence intervals that accompany the largest odds ratios reflect the low absolute incidence of leakage that constrains every study in this field, including our own.

Intellectual honesty requires equal attention to the discordant evidence, because not every analysis has found that low muscle mass predicts leakage. In a 2018 meta-analysis of 12 studies and 5337 patients, Sun and colleagues showed that sarcopenia defined by the third lumbar skeletal muscle index predicted longer hospital stay, higher overall morbidity, greater mortality, and more infections, yet it did not predict anastomotic leakage or intestinal obstruction [28]. More strikingly, the 2021 meta-analysis by Trejo-Avila and colleagues, which pooled 44 observational studies and 18,891 patients, reported that leakage occurred at a comparable rate in sarcopenic and non-sarcopenic patients, with a pooled odds ratio for leakage of 0.99 (95% confidence interval 0.72 to 1.36), even as the same analysis confirmed worse overall morbidity, mortality, and survival [29]. In the same vein, Looijaard and colleagues reported that computed tomography body composition was not consistently associated with outcome in older patients with colorectal cancer [30], and a recent 18-year cohort found that sarcopenia predicted long-term survival but not postoperative complications after gastric cancer surgery [31]. The apparent paradox, in which large pooled analyses detect no leakage signal while individual cohorts such as ours detect a strong one, is best explained by the dilution that occurs when heterogeneous studies are combined. Pooling across different muscle definitions, different anatomical compartments, different thresholds, and different operative contexts can average away an association that is real within a methodologically uniform single-centre sample. This tension does not weaken our finding so much as it defines the conditions under which the skeletal muscle index is most informative, and it argues for standardised measurement before the marker can be expected to perform consistently across institutions.

Several specific sources of heterogeneity deserve emphasis. First, the muscle compartment measured varies widely across studies, from the psoas alone, as used by Herrod and Batista [25,32], to the total skeletal muscle area at the third lumbar level that we employed, and Baracos has warned that the psoas is a flawed sentinel for whole-body muscle because it represents only a small and atypical fraction of total muscle mass [33]. Second, the threshold used to define a low index differs between studies, with many adopting sex-specific cut-offs while others, including the present study, apply a single data-driven value (specifically, our ROC-derived cut-off of 48.6 cm^2^/m^2^) for simplicity and comparability. Third, the operative context matters, because rectal anastomoses lie lower in the pelvis and carry a higher baseline leak rate than colonic anastomoses, so the leakage signal attributable to muscle mass can be confounded by the proportion of rectal cases in a cohort, and the surgical approach adds further variation, as illustrated by studies of laparoscopic resection in sarcopenic patients [34]. Fourth, muscle quantity and muscle quality are not interchangeable, and myosteatosis, the fatty infiltration of muscle that Lee and Kang demonstrated as carrying independent prognostic weight in colorectal cancer, may capture risk that a simple area-based index misses [35]. The methodological uniformity of our cohort, in which a single experienced radiologist measured the total lumbar muscle compartment on a single scanner platform and leakage was defined by a single international standard, plausibly sharpened the signal that pooled analyses tend to blur.

These operating characteristics define how the index should and should not be used. With a specificity of 38.2% and a positive predictive value of 10.0%, a low SMI cannot by itself identify the individual patient who will leak, and it should not drive a major surgical decision in isolation. Using the threshold to mandate a diverting stoma in every low-index patient, for example, would expose 269 of the 299 flagged patients to an intervention they did not need. The defensible interpretation is the converse, where a preserved index reassures, marking a low-risk subgroup, while a low index should prompt closer perioperative attention as one component of a broader multivariable assessment rather than a stand-alone trigger for action.

The work most directly comparable to ours remains that of van Kooten and colleagues, who examined computed-tomography-based preoperative muscle measurements as predictors of anastomotic leakage after oncological sigmoid and rectal resection and found that low muscle measures associate with leakage [26]. Our larger cohort independently corroborates that conclusion with a clear and significant effect size, and it does so using the total lumbar muscle compartment rather than the psoas alone. Taken together with the psoas-based reports of Herrod and Batista [25,32] and the multi-centre body composition analyses of Malietzis and Martin [16,36], our results consolidate the view that the muscle compartment visible on every staging scan encodes a clinically meaningful signal for anastomotic failure, provided that it is measured consistently and interpreted with attention to sex, anatomical level and muscle quality.

Viewed together, these recent reports describe a consistent direction of effect despite considerable variation in magnitude. Su and colleagues reported an odds ratio of 3.42 for postoperative complications in 387 laparoscopic colorectal cancer patients [13], while the meta-analyses of Keshavjee and colleagues, drawing on 40 studies and 13,422 patients, and of van Helsdingen and colleagues, drawing on 45 studies and 16,537 patients, confirmed that computed tomography body composition tracks postoperative outcomes after colorectal cancer surgery [11,12]. Tirnova and colleagues identified low muscle mass as an independent risk factor for major complications in older colon cancer patients [18], and the broader gastrointestinal surgical oncology meta-analysis of Simonsen and colleagues placed sarcopenia among the determinants of major postoperative complications [9]. Within this landscape, the leakage-specific studies are the most directly comparable to ours: van Kooten and colleagues linked low preoperative muscle measures to anastomotic leakage after sigmoid and rectal resection [26], and our adjusted odds ratio of 5.67 ranks among the largest reported for leakage specifically. That our estimate sits at the stronger end of the distribution most plausibly reflects two features of the present work, namely the use of leakage as a single discrete endpoint rather than a composite of complications, and the methodological uniformity of a single-centre cohort measured by one radiologist on one scanner platform. The convergence of these diverse populations, measurement protocols and analytic methods on the same direction of effect, even as the magnitude varies, is itself a strong argument that the signal we report is real rather than incidental.

Looking ahead, the most promising path lies in combining SMI with complementary body composition and clinical variables into an integrated risk model. Muscle radiodensity, visceral and subcutaneous adipose tissue, and the construct of sarcopenic obesity each contribute prognostic information that the SMI alone cannot capture, as visceral adiposity and sarcopenic obesity have each been associated with postoperative complications in colorectal and gastrointestinal cancer surgery [37,38,39]. Integrated body composition assessments that combine muscle and adipose measures have shown value beyond any single parameter [16,40]. Embedding such a model within the electronic record, fed automatically by artificial-intelligence segmentation of the staging scan, would allow the surgeon to receive a calibrated leakage probability before the patient ever reaches the operating theatre. The marginal cost of such a system is low precisely because the imaging already exists, and the potential benefit is the earlier deployment of nutritional support, prehabilitation and selective faecal diversion to the patients who stand to gain most.

The clinical pathway implied by our data is therefore pragmatic rather than radical. A surgeon reviewing a staging scan could note a low SMI, escalate preoperative nutritional optimisation and structured prehabilitation, lower the threshold for a defunctioning stoma in borderline anastomoses, intensify early postoperative monitoring for the signs of leakage, and document the elevated risk during informed consent. None of these measures is novel in isolation, but SMI offers an objective and reproducible trigger that replaces subjective impressions of frailty with a number anchored to the patient anatomy. In this sense, the value of SMI lies less in its stand-alone discrimination, which our area under the curve of 0.646 shows to be modest, and more in its ability to direct attention and resources toward a clearly defined high-risk subgroup.

Perhaps the most clinically useful property of SMI in our analysis was its negative predictive value of 98.2%. The high sensitivity of 90.9% combined with this negative predictive value means that a preserved SMI reliably identified patients at very low risk of leakage. In practical terms, a high SMI offered strong reassurance, whereas a low SMI flagged a group warranting heightened vigilance. The corresponding trade-off was a modest specificity of 38.2% and a low positive predictive value of 10.0%, which reflect the relatively low baseline incidence of leakage. A low SMI should therefore be read as a trigger for risk-mitigating strategies rather than as a deterministic predictor of failure. Such strategies might include preoperative nutritional optimisation, prehabilitation, a considered threshold for a defunctioning stoma, intensified postoperative surveillance, and a frank discussion of risk during informed consent.

The sex distribution of our SMI groups merits comment because it illustrates a methodological caution. The high-SMI group was predominantly male, which aligns with the well-established physiological reality that men carry greater muscle mass. This pattern argues for sex-specific reference thresholds in future work, in keeping with the sex-stratified cut-offs adopted by the major sarcopenia consensus groups [6,8]. A single threshold applied across both sexes, as used here for simplicity and for comparability with prior single-cut-off studies, may misclassify some patients, and the integration of sex-specific cut-offs represents a logical refinement. It is also notable that muscle depletion carries prognostic weight independent of body mass index, which helps explain why our cohort, overweight on average, still showed a strong muscle-related signal [41], and this body composition perspective underlies the so-called obesity paradox observed in cancer surgery [42].

From a health-systems perspective, SMI is unusually attractive because it is essentially free. Almost every patient with colorectal cancer undergoes staging computed tomography, so SMI can be extracted from images already acquired, without additional cost, contrast or radiation [43]. The growing availability of automated and semi-automated segmentation tools promises to embed SMI reporting directly into the radiology workflow, which would remove the labour barrier that has historically limited routine adoption. A measurement that demands no new scans, no new appointments, and no new expenses yet refines the prediction of a complication as serious as anastomotic leakage is precisely the kind of pragmatic biomarker that surgical practice needs. The economic case is reinforced by evidence that major surgery costs substantially more in the sarcopenic patient [44].

At the tissue level, the link between depleted muscle and a failing anastomosis is increasingly understood as more than a marker of general debility. Skeletal muscle functions as a secretory organ that releases myokines and helps regulate systemic immune and metabolic homeostasis, so its depletion may directly impair the coordinated inflammatory and proliferative phases of anastomotic healing rather than merely accompanying poor nutrition [10,17]. Sarcopenia also frequently coexists with a chronic catabolic and pro-inflammatory state, with hypoalbuminaemia and with reduced microvascular perfusion, each of which compromises collagen deposition and the early mechanical integrity of the suture line. Viewed through this lens, the patient with a low SMI enters the operation with a healing apparatus that is already operating near the limit of its reserve, and the technical perfection of the anastomosis cannot fully compensate for a host that lacks the biological capacity to seal it. This framework helps explain why a measurement of muscle bulk, distant from the bowel itself, can carry such a strong and independent signal for a complication of the bowel wall.

### 4.1. Strengths

Alongside its limitations, the study has notable strengths. The cohort was large and consecutive, which reduced selection bias within the eligible population and provided adequate statistical power to detect a clinically meaningful association despite the relatively low event rate. All muscle measurements were performed by a single experienced abdominal radiologist using a uniform protocol on a single scanner platform, which eliminated inter-observer and inter-scanner variability and lent the SMI values an internal consistency that multi-centre datasets often lack. The outcome was defined according to the widely accepted International Study Group of Rectal Cancer criteria, which anchors the leakage definition to an international standard and aids comparability with the broader literature. Finally, the analysis paired a clinically interpretable cut-off with a multivariable model, so that the findings translate readily into the bedside language of sensitivity, negative predictive value, and adjusted odds, instead of remaining confined to abstract statistical association.

### 4.2. Limitations

This study has several limitations. Its retrospective, single-centre design limits generalisability, and external validation in independent, preferably multi-centre cohorts is needed before the SMI threshold is adopted clinically. Several established risk factors for leakage, including tumour location, anastomotic level, neoadjuvant therapy, serum albumin, and operative variables, were not recorded in this retrospective dataset and could not be entered into the model; therefore, residual confounding by surgical factors cannot be excluded, and the independence of the index should be read within the limits of the available covariates, while the relatively small number of leakage events limited the number of covariates that could be modelled. The cut-off was derived within the cohort, and the moderate area under the curve indicates that the SMI is best used as one component of a broader preoperative risk assessment rather than as a stand-alone test. Finally, the measurement captured muscle quantity but not quality, and leakage was treated as a binary endpoint without severity grading or long-term outcomes; prospective, multi-compartment studies will help establish SMI within validated risk models. Leakage was assessed within the first 30 postoperative days in line with the standard definition; late leaks presenting beyond this window were not systematically recorded and may have been missed, although such events are uncommon. The cohort was restricted to undiverted anastomoses, as patients with a covering loop ileostomy were excluded; the findings therefore apply to patients managed without primary faecal diversion.

## 5. Conclusions

In this large, single-centre cohort, a low preoperative SMI independently increased the odds of anastomotic leakage almost six-fold after colorectal cancer surgery, and it acted as the only significant predictor among the clinical variables examined. A threshold of 48.6 cm^2^/m^2^ combined high sensitivity with an excellent negative predictive value, so that a preserved SMI reliably marked a large group of low-risk patients while a depleted SMI flagged those who deserve closer attention. Because SMI can be obtained at no additional cost or radiation from computed tomography images already acquired for staging, it represents a pragmatic and readily deployable adjunct to preoperative risk stratification. Multi-centre prospective validation with sex-specific thresholds, comprehensive clinical covariates, and graded leakage outcomes is now warranted to confirm these findings and define how SMI assessment can best be woven into routine surgical decision-making.

## Figures and Tables

**Table 1:** Baseline demographic, anthropometric and radiological characteristics of the cohort (n = 468).

Variable	n (%) or Mean ± SD	Median (Min to Max)
Anastomotic leakage, present	33 (7.1)	-
Anastomotic leakage, absent	435 (92.9)	-
Sex, male	299 (63.9)	-
Sex, female	169 (36.1)	-
Age, years	63.9 ± 13.3	66 (22 to 101)
Body mass index, kg/m^2^	26.9 ± 3.8	26.1 (17.9 to 51.1)
Height, cm	168.0 ± 7.1	169 (145 to 185)
Skeletal muscle index, cm^2^/m^2^	46.4 ± 11.1	44.2 (22.1 to 76.4)
Muscle surface area, cm^2^	127.6 ± 35.0	122.6 (55.6 to 247.4)
Measured volume, cm^3^	22.1 ± 17.4	15.5 (5.6 to 109.6)
Section thickness, mm	1.65 ± 1.1	1.50 (1 to 5)

SD, standard deviation. Skeletal muscle index and area were measured on axial computed tomography at the third lumbar vertebra.

**Table 2:** Diagnostic performance of the skeletal muscle index for predicting anastomotic leakage.

Metric	Value (95% CI)
Cut-off, cm^2^/m^2^	<48.6
Area under the curve	0.646 (0.599 to 0.688)
Sensitivity, %	90.9 (75.7 to 98.1)
Specificity, %	38.2 (33.6 to 42.9)
Positive predictive value, %	10.0 (8.9 to 11.2)
Negative predictive value, %	98.2 (94.9 to 99.4)
*p* value	0.002

CI, confidence interval. Performance derived from receiver operating characteristic analysis with the Youden-optimal threshold.

**Table 3:** Comparison of variables between the low and high skeletal muscle index groups.

Variable	Low SMI (<48.6) n = 299	High SMI (≥48.6) n = 169	*p* Value
Sex, female, n (%)	123 (41.1)	46 (27.2)	0.002
Sex, male, n (%)	176 (58.9)	123 (72.8)	
Anastomotic leakage, n (%)	30 (10.0)	3 (1.8)	0.0008
Age, years, mean ± SD	64.3 ± 12.9	63.0 ± 14.0	0.535
Body mass index, kg/m^2^	26.7 ± 3.6	27.2 ± 4.3	0.196
Measured volume, cm^3^	16.9 ± 12.9	31.2 ± 20.3	<0.001
Section thickness, mm	1.47 ± 0.9	1.96 ± 1.2	<0.001
Surface area, cm^2^	112.2 ± 21.5	154.9 ± 37.5	<0.001
Height, cm	167.7 ± 6.9	168.6 ± 7.3	0.178

SD, standard deviation; SMI, skeletal muscle index. The chi-square test or Fisher’s exact test, where appropriate, was used for categorical variables, and the Mann–Whitney U test was used for continuous variables.

**Table 4:** Multivariable logistic regression analysis of factors associated with anastomotic leakage.

Variable	Odds Ratio	95% CI Lower	95% CI Upper	*p* Value
Low SMI (<48.6)	5.671	1.692	19.007	0.005
Body mass index	0.913	0.815	1.023	0.117
Female sex	1.414	0.678	2.949	0.355
Age (per year)	1.008	0.981	1.036	0.571

CI, confidence interval; SMI, skeletal muscle index. The outcome was the presence of anastomotic leakage.

## Data Availability

The de-identified data presented in this study are available from the corresponding author upon reasonable request. The data are not publicly available because of privacy restrictions.

## Abbreviations

AUC, area under the curve; CI, confidence interval; CRC, colorectal cancer; CT, computed tomography; EWGSOP, European Working Group on Sarcopenia in Older People; ISGRC, International Study Group of Rectal Cancer; NPV, negative predictive value; OR, odds ratio; PPV, positive predictive value; ROC, receiver operating characteristic; SMA, skeletal muscle area; SMI, skeletal muscle index; STROBE, Strengthening the Reporting of Observational Studies in Epidemiology.
